# The complete mitochondrial genome of the hybrid of *Acanthopagrus schlegelii* (♀) × *Pagrus major* (♂) with phylogenetic analysis

**DOI:** 10.1080/23802359.2018.1501305

**Published:** 2018-10-25

**Authors:** Fei Zhu, Liangyi Xue, Chaofeng Jia, Zhiyong Zhang, Shuyin Chen, Zhiwei Zhang, Qian Meng, Ruijian Sun

**Affiliations:** a College of Marine Sciences, Ningbo University, Ningbo, China;; b Aquaculture and Genetic Breeding Laboratory, Marine Fisheries Research Institute of Jiangsu Province, Nantong, China;; c Jiangsu Key Laboratory for Genetics and Breeding of Marine Fishes, Nantong, China

**Keywords:** Hybrid sea bream, mitochondrial genome, next-generation sequencing

## Abstract

In this study, we firstly determined the complete mitochondrial DNA sequence of the hybrid of *Acanthopagrus schlegelii* (♀)×*Pagrus major* (♂) using the next-generation sequencing and Polymerase Chain Reaction-based method (PCR). The total length of the hybrid mitochondrial genome was identical to the female parent as 16,649 bp in length, which contained 13 protein-coding genes (PCGs), two ribosomal RNA genes, 22 transfer RNA genes, and one displacement loop locus (a control region). The overall nucleotide composition is: 28.0%A, 27.9%T, 27.9%C, and 16.2%G, with a total A + T content of 45.9%. This study discovered the 99.8% sequence identity between the hybrid and its female parent, which confirmed the maternal inheritance pattern followed by the mitochondrial genome of the hybrid. The complete mitochondrial genome sequence of this hybrid sea bream may provide a valuable and useful resource for population genetic study and monitoring, and as well as for further conservation effort on this species.

*Acanthopagrus schlegelii* and *Pagrus major*, both belong to Sparidae in the order Perciformes, which are widely distributed in the West Pacific coast from Japan, Korea to the East China Sea. They are the most important species commercialized for food in various areas (Chuen-Tan et al. 1996; Perez-Enriquez et al. 2001). As the hybrid generation has a faster growth and a better disease resistance, and there is little information of F1 genetic characteristics, we use the next-generation sequencing techniques and PCR-based method to study the complete mitochondrial genome of the hybrid sea bream (*A. schlegelii*♀ × *P. major*♂). The hybrid sea bream (*A. schlegelii*♀ × *P. major*♂) samples were obtained by artificial hybridization from Jiangsu Fish Breeding Base of Institute of Oceanology and Marine Fisheries, China. Total genomic DNA was extracted from these caudal fins by a Tissue DNA Kit (Aidlab Biotechnologies Co. Ltd, Beijing, China) following the manufacturer’s protocol.

The total length of the mitochondrial genome was identical to the female parent as 16,649 bp in length (Genebank, MH363710), which contained 13 protein-coding genes (PCGs), two ribosomal RNA genes, 22 transfer RNA genes, and one displacement loop locus (a control region). The overall nucleotide composition is: 28.0%A, 27.9%T, 27.9%C, and 16.2%G, with a total A + T content of 45.9%, respectively. Most of coding genes were encoded on the heavy strain (H-strand) except for *ND6* and eight *tRNA* genes, which were encoded on the light strain.

To determine the phylogenetic position of *A. schlegelii*♀ × *P. major*♂, it was conducted by using mitochondrial genomic data sets of this study and 14 other fish species as well as one outgroup species from the GeneBank database (Figure 1). Three primary branches were supported, among them, *A. schlegelii*, *A. schlegelii*♀ × *P. major*♂, *A. latus*, *Pagellus bogaraveo*, *Rhabdosargus sarba*, *Sparus aurata*, *Spicara maena*, *P. auriga*, *P. major*, *Parargyrops edita*, clustered into one branch, which 10 taxa all belong to Sparidae. Meanwhile, the hybrid of *A. schlegelii*♀× *P. major*♂ has a closer relationship with *A. schlegelii* (female parent) than *P. major* (male parent). The hybrid has a closer relationship and smaller genetic distance from its female parent (*A. schlegelii*) than its male parent (*P. major*), which can be explained by maternal inheritance characteristics of the mitochondria. *Lutjanus johnii*, *L. rivulatus*, *L. malabaricus*, *L. sebae* clustered into another branch, which four taxa belong to Lutjanidae. Except the previous two, *Takifugu rubripes* was an outgroup.

**Figure 1.**
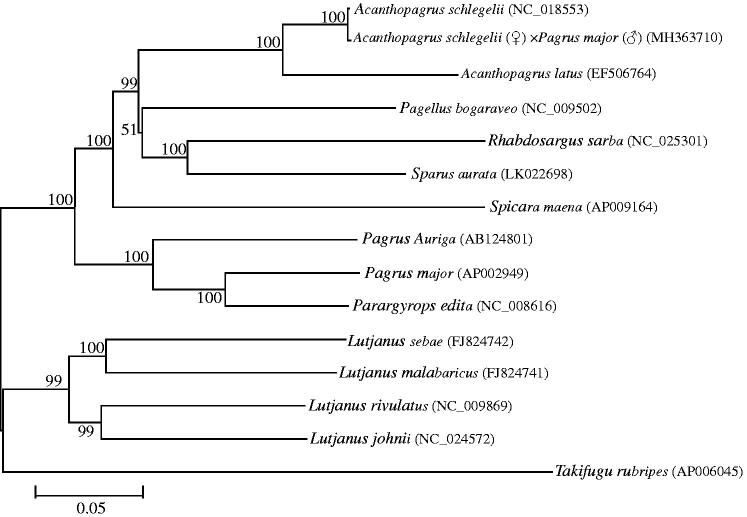
The NJ phylogenetic tree of Perciformes species. Numbers on each node are bootstrap values of 1000 replicates.

In summary, the present study first reported the complete mitochondrial genome of the hybrid sea bream *A. schlegelii* (♀) × *P. major* (♂). The circular molecule was 16,649 bp long and showed a typical vertebrate mitogenome structure. Phylogenetic analyses showed that mitochondrial genomes of *A. schlegelii* (♀) × *P. major* (♂) remain maternally inherited, which was consistent with the mitochondrial inheritance mechanism. The complete mitochondrial genome sequence of *A. schlegelii* (♀) × *P. major* (♂) provided an important dataset for a better understanding of the mitogenomic diversities and evolution in fish as well as novel genetic markers for studying population genetics and species identification.
